# Economic and educational factors played roles in the development of regional vision impairment in Shandong province, China

**DOI:** 10.1038/s41598-021-95906-7

**Published:** 2021-08-16

**Authors:** Lizhen Han, Jinzhu Jia, Lu Wang

**Affiliations:** 1 Department of Biostatistics, School of Public Health, Peking University, No. 38, Xueyuan Road, Haidian District, Beijing City, 100191 China; 2 Physical Examination Office of Shandong Province, No. 9 Yandong New Road, Lixia District, Jinan City, 250014 Shandong Province China

**Keywords:** Paediatrics, Public health

## Abstract

We analyze regional factors and spatial distribution of children's vision impairment in Shandong province, to explore the spatial changes brought by time and their influencing factors, so as to provide scientific basis for prevention of childhood vision impairment. This study covers five complete cross-sectional surveys from 2013 to 2017, involving about 29.24 million students. Spatial autocorrelation and hotspot analysis methods were used to analyze spatial features. The associated factors were analyzed by multinomial logistic regression. The vision impairment prevalence showed a trend of decreasing first and then increasing from 2013 to 2017, with slight changes. In terms of regional spatial differences, Weihai and Yantai have the highest VI rates in all years, and there was a large-scale spatial aggregation phenomenon. The southern low-value clusters, however, showed a trend of weakening from year to year. Further exploration revealed that economic factors and number of full-time teachers were verified as risk factors for regional vision impairment levels. The slight rebound of the prevalence of vision impairment and the high rate in the eastern and northern regions of Shandong province need more attention. It is suggested that relevant departments should focus on the influence of regional economic and educational factors when formulating relevant strategies.

## Introduction

Childhood is considered to be one of the most critical stages of growth and development, which has long been concerned from many aspects, such as the state, schools, families and individuals^1^. How to create a healthier growth environment for children in the current situation (increasingly complex living environment) is an urgent problem to be solved. Among them, vision issues (such as vision impairment (VI), myopia, amblyopia, etc.) are most common and prominent. As a window for one to understand and observe the world, the importance of eyes is self-evident. In China, a populous country that has liberalized the Two-Child policy, the prevalence of eye problems such as myopia in children and adolescents has been rising in recent years^2,3^. For example, the VI (distance vision impairment) rate with strong generality has rapidly increased almost 2.5-fold from 23.7% to 55% (1985–2014)^2^. From a global perspective, China also has a high prevalence of both myopia and low vision^4,5^. The vision impairment has become an urgent health problem in China, which requires cooperation of various departments.

Vision impairment refers to deficits in the ability of the person to perform vision-related activities of daily living. A vision impairment results when an eye condition affects the visual system and one or more of its vision functions^6^. It also reflects the burden of vision loss for the person. Studies have confirmed that there are many causes for the occurrence of VI, including heredity (e.g. macular dystrophies and retinitis pigmentosa), diseases (e.g. posterior segment (retinal) diseases, corneal degenerations and optic nerve head disease) and trauma, etc.^7–9^ Compared with normal sight children, the overall quality of life (QoL) of vision impaired children is relatively lower^10^. This is not only manifested in physical health and psychological aspects (e.g. depression^11^), but also in social relations and living environment^12^, and its impact will be lifelong. A recent study by Jones, N and other scholars found that vision impairment will affect the corresponding life skills (e.g. shopping and cooking) when it develops into adulthood^7^. In addition, visually impaired children will have a relatively lower level of participation in sports activities (the higher the degree of VI, the lower the level of participation)^7,13^. This phenomenon will not merely affect children's social interaction and exercise, but also give rise to a series of problems such as overweight and obesity^14,15^.

On the other hand, the relationship between vision impairment and economy is also inseparable. Research showed that for every 100% increase in gross domestic product (GDP), the risk of VI will rise by 20%, and the risk of moderate to severe VI will even increase by 27%^2^. Meanwhile, higher income level was significantly associated with VI in a gradient across severity of VI^16^. In China, the direct cost per patient due to VI is US$ 6988.6 ± 10,834.3 per year, of which 70% is direct medical expenses and only less than 30% can be reimbursed^17^. A recent German study also showed that VI and blindness have brought huge social burden (annual cost is about EU€49.6 billion)^18^.

Globally, the number of people with vision impairment of all ages reached approximately 285 million back in 2010, and that number increased to 2.2 billion by 2019^4,5^. Albeit the older age group still occupies the majority in general, the prevalence of younger group has increased even more rapidly^2^. Therefore, this article will study the evolution of childhood vision problems in Shandong province (about 100 million people), a representative province in China, based on the VI prevalence of children aged 6–12. The aim was to find out the long-term trend, regional distribution characteristics and associated risk factors through the analysis on schoolchildren with vision impairment from 2013 to 2017, so as to provide data support for the next policy adjustment and targeted preventive interventions by the government and relevant departments. Our ultimate goal is to create a healthier growth and living environment for children.

## Results

### Basic characteristics of research subjects

The sample was from the physical examination data of primary and secondary school students in Shandong province (2013–2017), and children aged 6–12 years old were selected as subjects (a total of 29 237 771, mean (SD) age: 9.06 (1.91) years; 53.96% were boys), which basically covers whole school children in Shandong province. Among them, the sample size in these 5 years were 1 864 241, 5 859 099, 6 685 362, 7 065 383 and 7 763 686 respectively. Basic information of children in each city can be found in Table 1.

**Table 1:** Information of children in different cities of Shandong province from 2013 to 2017.

City	Number of children (mean age (years))				
	2013	2014	2015	2016	2017
Binzhou	–^a^	249 557(8.91)	251 354(8.93)	252 737(8.97)	272 209(8.95)
Dezhou	35 673(9.00)	357 337(8.85)	360 762(8.82)	421 942(8.84)	441 156(8.90)
Dongying	48 955(8.95)	118 448(8.91)	131 950(8.93)	140 396(9.00)	144 435(9.01)
Heze	–^a^	870 954(8.66)	919 634(8.72)	953 536(8.80)	1 025 622(8.86)
Jinan	201 006(8.77)	272 641(8.76)	403 972(8.79)	435 378(8.85)	470 622(8.87)
Jining	17 802(9.38)	532 320(8.79)	581 734(8.82)	672 818(8.88)	722 942(9.06)
Laiwu	25 985(9.20)	66 054(9.12)	60 871(9.18)	73 363(9.06)	72 568(9.06)
Liaochen	1 007(9.24)	152 313(8.56)	453 943(8.63)	523 583(8.65)	591 048(8.79)
Linyi	188 051(8.74)	618 792(8.70)	776 428(8.74)	714 024(8.73)	1 049 007(8.83)
Qingdao	482 202(8.77)	550 205(8.74)	561 041(8.76)	601 326(8.92)	615 741(9.01)
Rizhao	74 876(8.76)	192 778(8.85)	192 202(8.94)	211 653(8.94)	210 700(8.97)
Taian	157 793(9.10)	337 878(9.04)	341 652(8.99)	346 422(9.02)	359 240(8.98)
Weihai	80 748(8.80)	129 262(8.78)	132 928(8.68)	142 225(8.86)	151 096(8.98)
Weifang	112 438(8.53)	570 382(8.72)	600 495(8.86)	613 485(9.04)	636 462(9.19)
Yantai	202 825(8.99)	329 707(8.93)	322 536(8.85)	342 520(9.02)	348 904(9.10)
Zaozhuang	7 478(8.40)	270 160(8.58)	323 092(8.55)	346 292(8.68)	374 907(8.85)
Zibo	227 402(8.92)	240 331(8.94)	270 768(8.94)	273 683(9.02)	277 027(9.08)

^a^The symbol (‘-’) represents a missing value.

### Basic information of vision impairment

**Figure 1:**
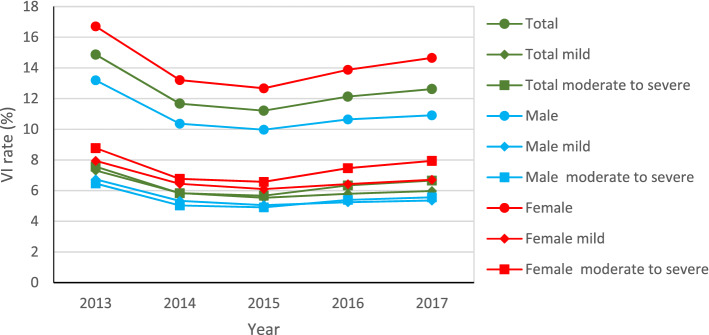
Trends in overall and gender-specific visual impairment (VI) among children in Shandong province from 2013 to 2017.

The total VI rates (T) for five years were relatively similar except for 2013 (14.87%), which fluctuated around 12% (11.21%—12.62%). The gap between different cities, however, was quite wide, especially in Weihai and Yantai, where the VI prevalence was the most prominent and has exceeded 20%. In general, VI rates presented a trend of decreasing first and then rising (2013–2017), like a concave curve (although no linear-by-linear association was detected, *p* = 0.707). In 17 different cities of Shandong province, the differences of VI prevalence in each year were statistically significant, and all showed the same trend (linear-by-linear association *p* < 0.001) except Weifang city (*p* = 0.214). In addition, the trends for the mild (M) and moderate-severe (M-S) VI rate were similar to the total (${r}_{T\&M}$= 0.980, *p* = 0.003; ${r}_{T\&M-S}$= 0.982, *p* = 0.003), and their results were very close (about 6%). The results were shown in Table 2 and Fig. 1.

**Table 2:** Vision impairment (VI) of children in different cities of Shandong province from 2013 to 2017.

City	Total VI rate (%)					*χ*^*2*^ value	*p*-value
	2013	2014	2015	2016	2017		
Binzhou	–^a^	11.04	9.32	11.01	8.58	1325.08	< 0.001
Dezhou	7.17	11.00	10.88	11.72	14.63	4448.80	< 0.001
Dongying	15.99	18.07	18.68	19.78	18.27	380.16	< 0.001
Heze	–^a^	5.85	5.75	7.21	8.05	5657.60	< 0.001
Jinan	13.09	13.83	13.38	13.97	14.38	287.43	< 0.001
Jining	18.23	10.12	10.70	11.10	10.86	1348.03	< 0.001
Laiwu	15.97	18.33	18.02	19.79	20.57	358.02	< 0.001
Liaochen	6.95	9.85	7.68	8.50	8.38	736.83	< 0.001
Linyi	9.51	7.67	8.49	9.48	10.36	3978.22	< 0.001
Qingdao	15.14	13.89	13.95	16.08	17.09	3453.35	< 0.001
Rizhao	14.03	13.54	12.01	10.57	10.24	1729.40	< 0.001
Taian	12.57	12.29	12.97	12.51	14.95	1427.22	< 0.001
Weihai	21.92	22.53	20.42	20.71	20.47	276.64	< 0.001
Weifang	12.27	12.73	12.28	12.22	12.59	102.39	< 0.001
Yantai	22.77	21.05	20.01	22.57	22.74	1129.04	< 0.001
Zaozhuang	7.23	9.53	9.13	9.38	11.39	1350.70	< 0.001
Zibo	14.75	15.62	14.96	16.14	18.12	1440.65	< 0.001

^a^The symbol (‘-’) represents a missing value.

From Fig. 1, we found that the trends of VI rates for boys (B) and girls (G) were similar to that of the whole over the past 5 years (${r}_{T\&B}$= 0.995, *p* < 0.001; ${r}_{T\&G}$= 0.993, *p* = 0.001). No matter the total VI prevalence, or the mild or moderate to severe VI rate, obvious gender differences were detected (female rates > male rates), and the differences were statistically significant (${\chi}_{T}^{2}$=69,727.572, ${\chi}_{M}^{2}$=18,642.317, ${\chi}_{M-S}^{2}$=49,944.350, *p* < 0.001). It is worth noting that the same results were obtained by comparing students from urban and rural areas separately (${\chi}_{urban}^{2}$=33,506.411, ${\chi}_{rural}^{2}$=39,188.863, *p* < 0.001). Besides, the single-sex curve clarified that the prevalence of mild and moderate to severe VI was similar from 2013–2017.

**Figure 2:**
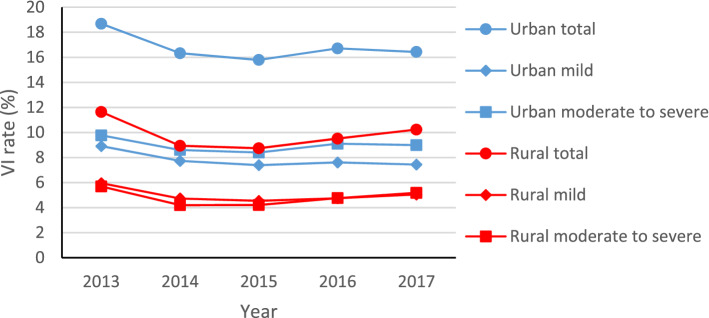
Trends in urban and rural visual impairment (VI) among children in Shandong province from 2013 to 2017.

Compared with rural areas, urban regions had a higher VI prevalence, such as the overall rate (${\chi}_{T}^{2}$=313,936.581, *p* < 0.001), this gap was maintained at more than 6% every year. Meanwhile, the urban–rural gap in mild and moderate-severe morbidities were also significant (${\chi}_{M}^{2}$=96,834.083, ${\chi}_{M-S}^{2}$=205,497.682, *p* < 0.001). However, in contrast, the upward trend of urban areas has declined in 2017. The results were shown in Fig. 2.

**Figure 3:**
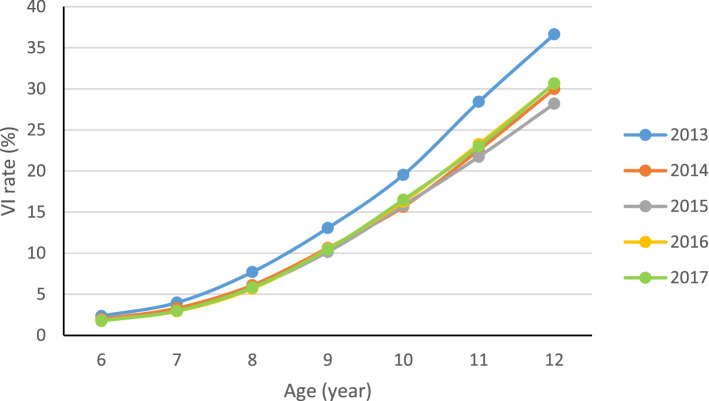
Age trends of visual impairment (VI) among children in Shandong province from 2013 to 2017.

In terms of age, there was a strong linear-by-linear association between VI prevalence and age (e.g. ${r}_{2015}$= 0.960, *p* = 0.001). The results (Fig. 3) demonstrated that the trends in each year were relatively consistent, showing a trend that the prevalence of VI increases with age (from about 2% at the age of 6 to 30% in 12 years old), and the difference between ages was statistically significant (${\chi}_{T}^{2}$=2,409,075.899, *p* < 0.001). Among them, the rise was more dramatic in 2013, while that in the other 4 years was relatively similar.

### Spatial analysis results

**Figure 4:**
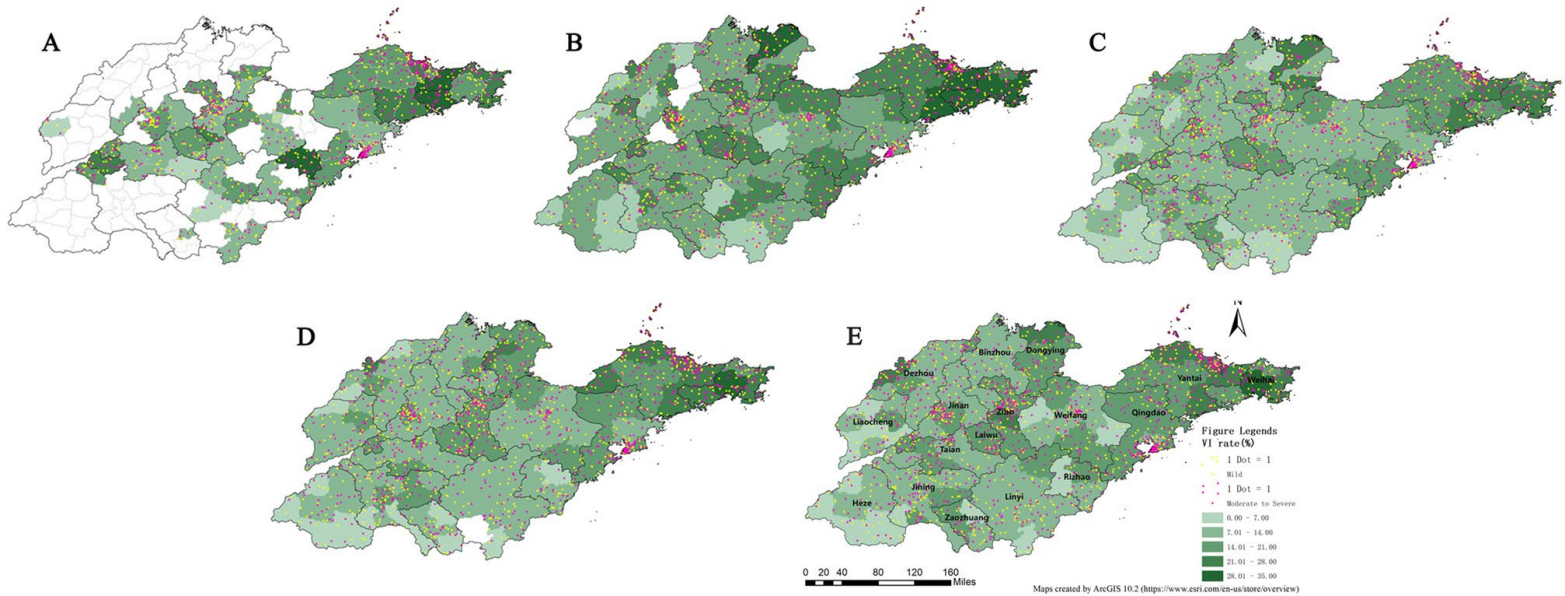
Spatial distribution of total visual impairment (VI) among children in Shandong province from 2013 to 2017. Figure Legends: Labels A to E represent the spatial distribution maps of each year from 2013 to 2017.

The results of spatial analysis showed that from 2013 to 2017, the regions with high prevalence of VI among children aged 6–12 years in Shandong province were mainly concentrated in the eastern peninsula and the northern area. Likewise, the prevalence in the central region was slightly higher than that in surrounding counties. It is worth noting that two new high-value districts were added in 2017: the western (parts of Dezhou) and central (parts of Zibo) regions, and their prevalence were significantly higher than those in previous years. The results were shown in Fig. 4.

Focusing on the regional distribution of mild and moderate-severe VI (yellow and purple dots) in these maps, it was found that the aggregation phenomenon was prominent in the northeast of the peninsula and areas around Jiaozhou Bay. In addition, the cluster of central counties has become more and more distinct with the passage of time.

Further, this study analyzed the spatial relationships that may exist between regional differences in overall VI rates. The results (Table 3) illustrated that there was spatial aggregation of VI in Shandong province.

**Table 3:** Spatial autocorrelation of vision impairment (VI) among children in Shandong province from 2013–2017.

Year	Moran’s I index	Variance	*z*-score ^a^	*p*-value
2013	0.40	0.01	4.76	< 0.001
2014	0.56	0.01	10.57	< 0.001
2015	0.53	0.01	10.32	< 0.001
2016	0.59	0.01	11.44	< 0.001
2017	0.53	0.01	10.26	< 0.001

^a^|*z*|> 2.58 indicates that the probability of randomly generating this clustering pattern is less than 1%.

Based on the spatial aggregation characteristics of VI in Shandong province from 2013 to 2017, the cluster map was presented after hotspot analysis. In 2013, although data were relatively scarce, a large range of high-value aggregation areas were still detected in the eastern part of Shandong peninsula (Weihai, Yantai), while the cold spot region was mainly concentrated in the central part. The distribution characteristics in the following four years were relatively consistent, and clusters with high/low values were located in the eastern/southern regions. In addition, in some years, a small number of counties in the north/west (hot/cold spots) also showed aggregation.

**Figure 5:**
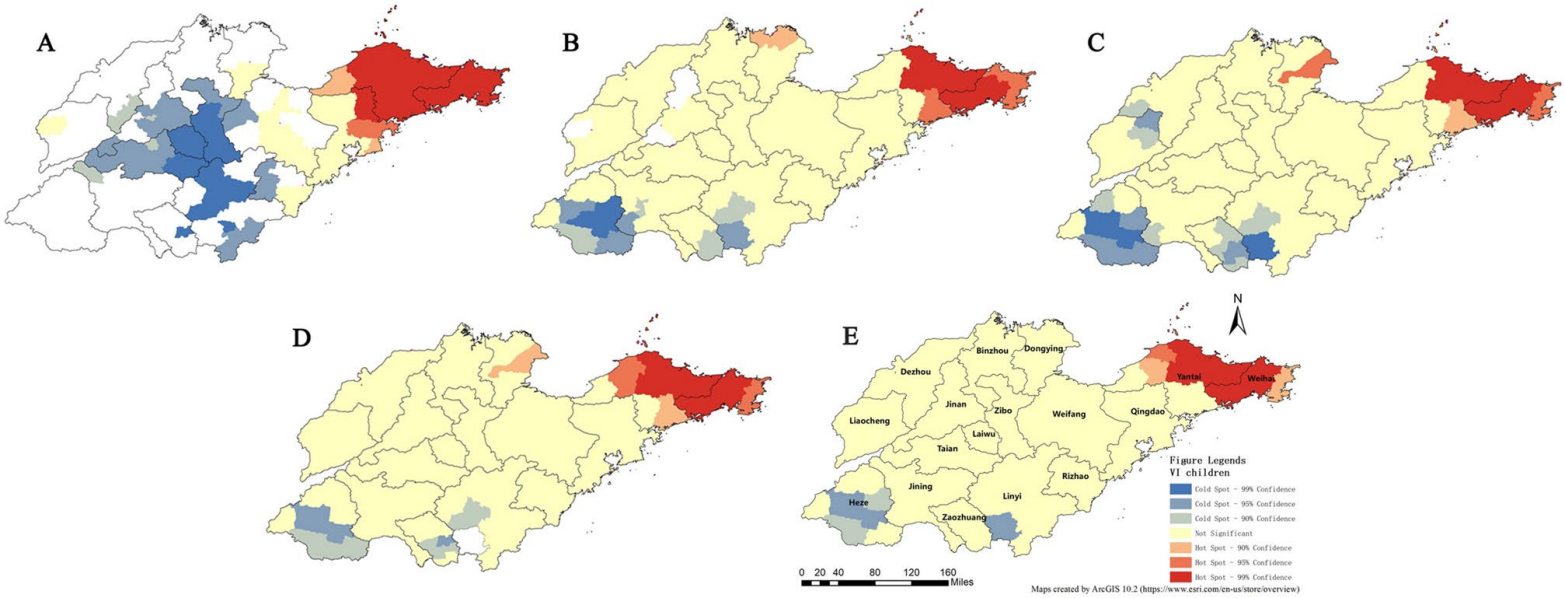
Spatial aggregation of visual impairment (VI) among children in Shandong province from 2013 to 2017. Figure Legends: Labels A to E represent the spatial cluster maps of each year from 2013 to 2017.

Combined with the maps of various years, the range of hot spots has been fluctuating slightly, while the accumulation of cold spots has shown a trend of weakening year by year, especially in Zaozhuang and Linyi cities. The results were shown in Fig. 5.

### Influencing factors analysis

After the collinearity diagnostics, five types of regional economic associated factors and one social influencing factor (including 137 counties in Shandong province): gross domestic product (GDP), the growth rate of GDP (GRGDP), general public budget expenditure (GPBE), total retail sales of consumer goods (CGTRS), per capita disposable income of rural households (RPCDI) and number of full-time teachers (FTT) were included in the multinomial logistic regression model. Among them, except GRGDP, the rest variables were weighted by regional population. Moreover, economic variables were recorded in units of CN¥1000 per capita (approximately equal to US$142.27), and the unit of FTT was 1 person.

Studies have confirmed that RPCDI, GRGDP and FTT were risk factors for regional VI level. RPCDI played a role in promoting the "development" of regional VI in different grades, while the latter two indexes only worked in the highest grade (compared with the level 1). Besides, CGTRS was also verified as a risk factor (only in level 3). Nevertheless, GDP and GPBE showed no statistically significant effect on the degree of VI. The results were shown in Table 4.

**Table 4:** Correlation between regional associated factors and vision impairment (VI) of children in Shandong province from 2013 to 2017.

Regional VI level	Factors	*β*	S.E	Wald	*p*	Exp*(β)*	95% CI for exp(*β*)	
							Lower bound	Upper bound
2	Intercept	-2.853	1.257	5.149	0.023			
	GDP	0.014	0.010	1.813	0.178	1.014	0.994	1.035
	GRGDP	0.017	0.027	0.395	0.530	1.017	0.964	1.074
	GPBE	0.045	0.107	0.174	0.676	1.046	0.848	1.289
	CGTRS	-0.001	0.023	0.004	0.950	0.999	0.955	1.044
	FTT	-0.001	0.006	0.047	0.829	0.999	0.987	1.011
	RPCDI	0.267	0.109	6.035	0.014	1.305	1.055	1.615
3	Intercept	-6.998	1.424	24.137	0.000			
	GDP	0.018	0.011	2.850	0.091	1.018	0.997	1.040
	GRGDP	0.006	0.032	0.035	0.851	1.006	0.945	1.070
	GPBE	0.014	0.111	0.017	0.897	1.014	0.817	1.260
	CGTRS	0.048	0.023	4.516	0.034	1.050	1.004	1.097
	FTT	0.001	0.008	0.008	0.929	1.001	0.986	1.016
	RPCDI	0.414	0.118	12.345	0.000	1.514	1.201	1.907
4	Intercept	-11.221	1.940	33.459	0.000			
	GDP	0.013	0.012	1.149	0.284	1.013	0.990	1.036
	GRGDP	-0.022	0.040	0.302	0.583	0.978	0.905	1.058
	GPBE	0.030	0.118	0.063	0.802	1.030	0.818	1.297
	CGTRS	0.028	0.026	1.236	0.266	1.029	0.979	1.081
	FTT	-0.001	0.012	0.005	0.943	0.999	0.976	1.022
	RPCDI	0.681	0.146	21.820	0.000	1.976	1.485	2.630
5	Intercept	-20.582	3.342	37.939	0.000			
	GDP	0.016	0.014	1.330	0.249	1.016	0.989	1.045
	GRGDP	0.116	0.055	4.345	0.037	1.123	1.007	1.252
	GPBE	-0.001	0.133	0.000	0.991	0.999	0.769	1.297
	CGTRS	0.021	0.033	0.419	0.518	1.022	0.958	1.090
	FTT	0.049	0.010	25.675	0.000	1.051	1.031	1.071
	RPCDI	0.932	0.221	17.738	0.000	2.540	1.646	3.919

## Discussion

As a large country with a population of 1.4 billion, China has an equally large number of children. Since the Two-Child policy was completely liberalized in early 2016, the number of newborns has increased significantly. Meanwhile, the VI prevalence is also continuously rising^2^. In order to better deal with children's vision impairment and provide them with a healthy growth environment, this study selected children from Shandong province as the research subjects, based on comprehensive demographic and economic indicators (domestic population ranking: 2nd, economic ranking: 3rd).

In terms of gender, statistically significant differences were detected. Whether the overall VI rate or mild, moderate-severe VI rates, the results of female group were significantly higher than those of male group. This is consistent with previous research results^19–21^. Although there is no direct evidence to prove the causes of this difference, according to other studies, we guessed that it may be affected by the following factors: We think the most important factor is that Chinese girls tend to be gentle and quiet under the traditional concept. Whether in study or sports, girls will spend more time indoors than boys. Furthermore, less outdoor activities will aggravate the degree of myopia^22^. Meanwhile, the higher prevalence of dry eye syndrome in women may be one of the reasons^23^. A hypothesis about hormone action may be another reason (although it is more reflected in the adolescent and older population)^24^. In the case of myopia, for example, women were mainly affected by follicle stimulating hormone and luteinizing hormone, while men were influenced by luteinizing hormone and testosterone^25^. In addition, the interaction between sex and steroidogenesis enzyme genes has also been proved to be a regulator of sex hormone metabolism and high myopia risk^26^.

At the age level, there was a strong positive correlation between VI rate and age. With the increase of age, the prevalence of VI among children also rose significantly. Multiple studies have confirmed this result^19,27^. When children are young, their eyeballs are smaller and their axes are shorter. The eyes are maintained in a state of hyperopia and have a certain "hyperopia reserve". After that, with the growth of children and the influence of various factors (such as increased schoolwork burden, more screen time, etc.), hyperopia reserve is consumed prematurely, and VI problems gradually become prominent and rapidly increase, like myopia. In addition, the higher school year also plays a negative role in visual development (e.g. study and exam pressure, reduced outdoor activity time)^28^.

The study found that the VI rate of children in Shandong province from 2013 to 2017 showed a flat U-shaped trend, with a slight decrease and increase. Considering that the data for 2013 only covered counties with better economy (the economy has a positive effect^16^), it has a high detection rate. After exclusion, there was an overall slow upward trend. Compared with previous study^2^, there were obvious differences. The reason for this phenomenon may be related to the gradual steady increase in children's living environment and economic factors in recent years, so the increase in VI rate has slowed down. Further analysis revealed that the prevalence of VI was higher in cities than that in counties. Albeit the economic gap between urban and rural areas in China is narrowing, "faults" still exist. Urban children living in a pleasant environment have more opportunities to contact with electronic products and even become addicted to them. Affected by this, compared with rural children, sedentary lifestyle of urban students tends to be normalized, and their visual condition also deteriorates^29,30^.

Through the integration of the annual distribution and the spatial aggregation maps, the high-value aggregation in the eastern part of the peninsula has been confirmed. Although the exact cause is unknown, combined with previous studies, the preliminary judgment is that it stems from the association between VI and overweight and obesity^31^—mainly influenced by sports activities^15,29^. Likewise, previous research on obesity in the same population found that the high prevalence area was exactly consistent with the above results^32^. Initial speculation suggested that children in Yantai and Weihai cities may have less time for physical exercise. The relationship between VI, exercise, overweight and obesity is like a two-way closed cycle, which interacts and affects each other (i.e. children with VI have less physical activity, and sedentary lifestyle will lead to high prevalence of obesity, and vice versa). Moreover, the influence of economy and day length cannot be ignored. Research by Cui, Dongmei and other scholars pointed out that axial growth and myopia progression will decrease with the increase of day length^33^.

From 2014–2017, the low-value aggregation range showed a narrowing feature (although the change was slight), and it was mainly concentrated in the southern areas. In this aspect, economic changes and the above-mentioned day length are mainly considered: The economic level of the southern region is lagging behind in all years, and the side effects brought by technology (e.g. massive open online course (MOOC), multimedia class, etc.) have slightly weaker impact on the students. On the other hand, the regional economy was still on the rise, which also explains the shrinking of the aggregations to some extent.

If subdivided by gender, the distribution characteristics (including total, mild and moderate to severe VI) were highly consistent with the overall, and the results for boys and girls were similar. Therefore, this article does not further explore the gender differences in regional distribution.

This study also included a variety of representative regional influencing factors, in order to conduct a more comprehensive discussion on the reasons of the above problems. After adjusting for confounding, through multiple logistic regression analysis, a total of four risk factors were detected. They were: RPCDI, GRGDP, CGTRS and FTT. When the regional VI level reached the fifth grade, they played the most significant role (except CGTRS). This means that compared with the underdeveloped areas, regions with higher RPCDI, GRGDP and FTT were more likely to develop into the regions with higher VI rate. Combining the above regional differences, we found that there were inextricable links between the economy and VI. When the regional economy and per capita disposable income have increased significantly (especially in the underdeveloped rural areas), the increase in purchasing power has greatly enhanced the affordability and consumption of electronic products, LED lamps and other items, and has accelerated the popularity of smart phones and tablets among young people, thus making it easier to form an unfavorable eye-using environment. Combined with the effects of long-term near work^34,35^ and harmful lights^36^, it is not surprising that the prevalence of VI was relatively high. Moreover, the risk effect of CGTRS was also manifested in the above aspects.

Children, as a group with low self-control, have difficulty making correct judgments about external events on their own. Meanwhile, early referral of children with vision loss is critical to ensure accurate diagnosis and prompt treatment of any modifiable aspects of the condition^37^. Therefore, the outcome will directly depend on the appropriate interventions taken by families (parents) and schools (teachers).

As another risk factor, the number of full-time teachers should also receive more attention from the departments. This study found that the risk of a region with abundant teacher resources evolving into an area with high VI level was 1.051 times that of a region with low VI level. The schoolwork pressure caused by teachers may have contributed to this result. Although this problem has been improved in primary schools, it can't be ignored due to the difficulty of entering a higher school (limited enrollment quota). Meanwhile, as the paramount part of the teaching process, teachers' behaviors, attitudes and professional teaching methods will affect children's health^38–40^. The prevalence of myopia among Chinese students without spectacles (the effect of frustration, discrimination, etc.) is to some extent accelerating the deterioration of vision^41,42^. The teacher workforce is growing, but its capacity is not comprehensive enough. Although teachers have a positive attitude toward children with VI^43^, they are generally unable to provide timely professional assistance to children with visual impairments (or they are ill-equipped to do so). In the context of Chinese parents' emphasis on traditional education, the burden on children is heavy, and teachers should further optimize their behavior and attitude to create a healthy environment for children's growth. However, the specific reasons for this result is still unclear. We suggested that future research should focus more on the teachers, so as to further explore the specific causes of this association.

In response to the existence of these problems, the Ministry of Education of the People's Republic of China and other departments promulgated a new implementation plan in 2018 (*Implementation plan for comprehensive prevention and control of myopia among children and adolescents*^44^). It provided comprehensive guidelines for families, individual students, schools, medical institutions and relevant departments. Among them, at the school level: The recommendations include academic load, physical exercise (more than one hour of physical activity time per day while at school), school hygiene and health education, and regular vision monitoring. The requirements stated that the implementation of vision care measures such as eye exercises should be adhered to, and that the environment (tables and chairs, lighting and illumination, etc.) and the amount of homework for different grades should also be regulated. Both at the family and school levels, the use of electronic products should be strictly controlled (paper homework, less than 30% of total class time for electronic product applications, etc.). In addition, it also set regulations for teachers, including the teaching and supervision of correct reading and writing posture, etc. When teachers notice signs such as students' inability to read the blackboard and frequent eye rubbing, they are required to keep track of their vision.

The introduction of these regulations has gone a long way toward alleviating the VI situation for children, but true effectiveness still needs to be ensured through comprehensive implementation. We suggested that the relevant departments should continue to improve the corresponding supervision and incentive mechanisms in order to more effectively put these measures in place. Particular emphasis should be placed on teacher-oriented health education training so that they can help children with VI in a competent, timely and professional manner^43,45^.

This is the first systematic study on the vision impairment of children aged 6–12 in Shandong province from 2013 to 2017, and the differences among them were expounded from various aspects. The sample involved in the study is huge, and the results are highly credible and regionally representative. Nevertheless, this research also has some limitations: 1) The year covered by this study is only 5 years, and the data in 2013 are missing a lot. Due to the limited conditions, the data in other years cannot be supplemented, so the results of trend analysis are for reference only. 2) The large-scale health examination (census) involved in the study was limited by conditions that prevented measurement of children's presenting vision, so the representativeness of the real-life vision as reflected by the vision impairment in the results obtained needs to be further improved. 3) The influencing factors were not comprehensive enough. Family, school and other related associated factors^20,35,46^ were not included in this research, and the effects by their interregional differences could not be verified. The above problems need further research.

## Methods

### Data source

The sample came from students’ physical examination data from September to December every year in Shandong province, totaling 5 years (i.e. 2013–2017). After adjusting for age and gender, the missing data and the error records beyond the scope of the visual chart (totaling 430,421) were eliminated. A total of 29 237 771 schoolchildren aged 6–12 years were included (person-time), covering whole primary schools in all 17 cities (137 counties) in Shandong province. The sample sizes in this data for different years ranged from 1 864 242 to 7 763 686, and the annual girl to boy ratio and urban to rural ratio approximately equaled 1:1.2 and 1:1.7, respectively.

### Measurement methods

According to the *Measures for the administration of health examination of primary and middle school students*^47^ and the *Measures for the implementation of health examination management for primary and middle school students in Shandong province*^48^, the National Health Commission of the People’s Republic of China, the Ministry of Education of the People’s Republic of China and the Shandong Provincial Education Department stipulate that students in school should have a physical examination every school year. This program was organized by the health administrative department at or above the county level, with rigorous training covering all medical professionals who participated in physical examination. All experimental protocols were approved by the Health Commission of Shandong Province and its subordinate institution (Physical Examination Office of Shandong Province) and all physical examination methods were carried out in accordance with the *Physical examination methods for primary and middle school students in Shandong province*^49^. In addition, the informed consent provided by the Physical Examination Office of Shandong Province was delivered to the student's parents through schools’ teachers. After the examination, students and parents will receive feedback in the form of reports.

According to the requirements of *Health examination methods for primary and secondary schools in Shandong province*, all physical examinations procedures were carried out in accordance with regulations and using standardized professional instruments. The data involved in this study were obtained through physical measurements and did not involve laboratory examinations.

Visual acuity (VA) was measured using the standard for logarithmic visual acuity charts (optotypes: tumbling Es, in line with the national standard GB11533—2011, International Classification for Standards (ICS) 13.100)^50^. Students need to keep the naked eye to participate in the test. Due to population size, human, material and financial resources, it was not possible to collect data on individual's presenting distance visual acuity in the census, and in conjunction with the recommendations in the *World report on vision* (that vision without spectacles or contact lenses is more appropriate for studying the entire population with vision impairment)^5^, unaided distance visual acuity was used in this study. During vision inspection, students should maintain a distance of 5 m from the instrument, and the sight line of the tested person should be consistent with the height of the visual chart (line "5.0"). Then, the left eye and the right eye are covered by the eye mask in turn, and the VA of the corresponding eye was detected. The reading of the smallest line that can be seen clearly by the subject was the VA of the relevant eye, and the value (decimal record) was recorded.

### Judging criteria for vision impairment

All children had been diagnosed with vision impairment according to the 11th International Classification of Diseases (ICD-11) issued by World Health Organization (WHO) in 2018^6^. According to the actual situation in China (i.e., the visual chart used in children's physical examination does not involve the detection range of less than 6/60) and previous studies^2,51^. In this study, vision impairment (distance vision impairment) was divided into mild and moderate-severe categories. Mild VI was defined as unaided VA of worse than 6/12 (logMAR > 0.3, decimal < 0.5) and that of equal to or better than 6/18 (logMAR ≤ 0.5, decimal > 0.3) in the worse eye, moderate to severe VI was defined as unaided VA worse than 6/18 (logMAR > 0.5, decimal < 0.3) in the worse eye.

### Other data

In terms of influencing factors, seven regional associated factors (obtained from the *Shandong Statistical Yearbook* over the years) were included, covering gross domestic product (GDP), the growth rate of GDP (GRGDP), general public budget expenditure (GPBE)—state financial expenditures that are spent on public services, education, medical care and social security, etc., total retail sales of consumer goods (CGTRS)—sales amount of physical commodities for non-production and non-business purposes (including catering services), number of full-time teachers (FTT)—weighted by the number of students in the region, representing the number of teachers per thousand students, and per capita disposable income of rural households (RPCDI). Meanwhile, in order to effectively reduce the impact of population differences in different areas, this study weighted the regional associated factors by their population (except GRGDP). In addition, based on the annual VI prevalence range and the effect of mapping, the regional VI level was divided into 5 grades from low to high (ranging from 0 to 35%, with every seven percentage points recorded as a level).

### Statistical analysis

Raw data was extracted through SQL Sever2017, and SPSS 22.0 was used to analyze the data. The measurement data were described by the mean (standard deviation), and t-tests and linear correlation were used for inter-group comparison. The counting data was expressed by the rate (%), and the comparative analysis between groups was tested by chi-square tests. Furthermore, multinomial logistic regression analysis was used to analyze population-weighted associated factors. A probability level of *p* < 0.05 represented the result with statistical significance.

### Spatial analysis

ArcGIS 10.2 software (https://www.esri.com/en-us/store/overview) was used to analyze spatial distribution, regional variation differences, etc. In light of the distribution range of VI rates, this study artificially divided it into 5 levels. Besides, in order to find out whether there is spatial clustering and its variation trend in the Shandong province, spatial autocorrelation (Global Moran's I) and hotspot analysis (Getis-Ord Gi*) were applied to this study.

Spatial autocorrelation (Global Moran's I) is a method for measuring spatial autocorrelation based on element locations and element values, which is used to evaluate whether the pattern expressed is clustered, dispersed, or random. It evaluates the significance of data results by calculating the Moran's I index value, *z*-score and *p*-value. If the index in the dataset tend to cluster spatially (aggregation of similar values), the Moran's index will be positive. Conversely, the index will be negative. If positive cross-product values balance negative cross-product values, the index will be near zero^52^.

The clustering of high and low values was obtained through hotspot analysis (calculation the Getis-Ord Gi* statistic for each feature in a dataset). After a partial sum of a feature and its neighbors was compared with the sum of all features, if the local sum was significantly different from the expected local sum that it cannot be randomly generated, a statistically significant z-score will be resulted. Based on the resultant *z*-scores and *p*-values, the location where the high-value or low-value elements cluster in space can be illustrated.

The Getis-Ord local statistic is given as:

$${G}_{i}^{*}=\frac{\sum_{j=1}^{n}{w}_{i,j}{x}_{j}-\overline{X }\sum_{j=1}^{n}{w}_{i,j}}{\mathrm{S}\sqrt{\frac{\left[\begin{array}{c}n\sum_{j=1}^{n}{w}_{i,j}^{2}-\left(\sum_{j=1}^{n}{w}_{i,j}\right)\end{array}\right]}{n-1}}}$$

where ${x}_{j}$ is the attribute value for feature $j$, ${w}_{i,j}$ is the spatial weight between feature $i$ and $j$, and:

$$\overline{X }=\frac{\sum_{j=1}^{n}{x}_{j}}{n}$$

$$S=\sqrt{\frac{\sum_{j=1}^{n}{x}_{j}^{2}}{n}-{\left(\overline{X }\right)}^{2}}$$

The ${G}_{i}^{*}$ statistic returned for each feature in the dataset is the z-score. For statistically significant positive z-scores, the larger the z-score is, the more intense the clustering of high values (hot spot). The opposite clustering method is called cold spot^53^.

### Patient and public involvement

No patients or public were involved in this study.

### Ethics approval and consent to participate

This study was exempted from need of ethical approval by the Research Ethics Committees of Shandong Center for Disease Control and Prevention. Ethics approval was not available in this study because we did not include any data of students’ personal information, including name, identity information, address, telephone number, etc. None of the authors in this study had access to identifying patient information during the analysis of the data. This study only showed the secondary aggregated data on county-level, therefore, waived off ethical approval. However, informed consent was still included in the implementation of this physical examination.

### Consent of publication

Not applicable.

## Conclusions

In conclusion, there was a significant phenomenon of high prevalence and aggregation of vision impairment in the eastern region of Shandong province, and economic and educational factors have played a certain role in the development of regional vision impairment. Relevant departments should be alert to the negative effects brought about by rapid economic growth. Based on the findings presented by each region, it is recommended that the relevant departments in each region should further refine their policies by integrating their own characteristics under the broad framework (*Healthy China 2030*, *Healthy China Action*, etc.) to improve the VI status of children (especially Weihai and Yantai). In addition, it is necessary to promote the reform and implementation of quality-oriented education (in terms of academic load, physical exercise and health education, etc.), as well as the application and popularization of assistive technologies^54^, and continuously improve monitoring mechanisms. Particular emphasis should be placed on education and training (e.g., professional knowledge and skills) for parents and teachers. Prevention and treatment should be combined to comprehensively improve the living environment of children and reduce the VI rate.

## Data Availability

The datasets generated and/or analyzed during the current study are not publicly available due the data is confidential, but are available from the corresponding author on reasonable request.
